# A Neural Network Framework for Validating Information–Theoretics Parameters in the Applications of Acoustic Emission Technique for Mechanical Characterization of Materials

**DOI:** 10.3390/ma16010300

**Published:** 2022-12-28

**Authors:** Claudia Barile, Giovanni Pappalettera, Vimalathithan Paramsamy Kannan, Caterina Casavola

**Affiliations:** Dipartimento di Meccanica, Matematica e Management, Politecnico di Bari, Via Orabona 5, 70125 Bari, Italy

**Keywords:** acoustic emission, CFRP, Lempel–Ziv complexity, k-means, data clustering, deep learning, CNN

## Abstract

A multiparameter approach is preferred while utilizing Acoustic Emission (AE) technique for mechanical characterization of composite materials. It is essential to utilize a statistical parameter, which is independent of the sensor characteristics, for this purpose. Thus, a new information–theoretics parameter, Lempel–Ziv (LZ) complexity, is used in this research work for mechanical characterization of Carbon Fibre Reinforced Plastic (CFRP) composites. CFRP specimens in plain weave fabric configurations were tested and the acoustic activity during the loading was recorded. The AE signals were classified based on their peak amplitudes, counts, and LZ complexity indices using k-means++ data clustering algorithm. The clustered data were compared with the mechanical results of the tensile tests on CFRP specimens. The results show that the clustered data are capable of identifying critical regions of failure. The LZ complexity indices of the AE signal can be used as an AE descriptor for mechanical characterization. This is validated by studying the clustered signals in their time–frequency domain using wavelet transform. Finally, a neural network framework based on SqueezeNet was trained using the wavelet scalograms for a quantitative validation of the data clustering approach proposed in this research work. The results show that the proposed method functions at an efficiency of more than 85% for three out of four clustered data. This validates the application of LZ complexity as an AE descriptor for AE signal data analysis.

## 1. Introduction

Identification, localization, and characterization of damage is one of the major applications of Non-destructive Evaluation (NDE) techniques. Among them, the passive NDE techniques are predominantly used in mechanical characterization of materials and structures under different loading conditions. The Acoustic Emission (AE) technique is one such passive NDE tool, which is capable of in-line characterization of material/structure under its entire loading history [1].

When a material is strained beyond its elastic limit, the elastic strain energy stored within the material is released. Some of the released energy propagates through the material as elastic waves [2,3]. This phenomenon is known as Acoustic Emission. Acquiring these elastic waves using contact-based or contact-less sensors and analysing them forms the basis of AE technique.

The AE technique has proved its formidability in assessing failure modes and damage progressions in complex materials such as Fibre Reinforced Polymer (FRP) composites. Over the last decade, the development of Artificial Intelligence has complemented the exponential growth of AE technique. In order to complement this growth, several new parameters are extracted from the recorded AE waveforms for analysis. Typically, basic descriptors of acoustic waveforms such as peak amplitude, number of counts, risetime, rise angle, energy, etc., are used for analysis [3,4,5,6,7,8,9,10]. Most of these time-dependent parameters are affected by sensor characteristics and the choice of the sensor and data acquisition system. Therefore, the search for a statistical parameter which is independent of the hardware characteristics of the acquisition system is prevalent. In recent years, information–theoretics parameters such as entropy and complexity indices have been used in AE analysis. In this research work, the complexity index introduced by Lempel and Ziv [11], commonly known as Lempel–Ziv complexity (LZ complexity), is explored. 

Researchers have proven the applicability of LZ complexity for analysing different types of time-series data acquired from biomedical to engineering applications. In the authors’ previous research work, the applicability of LZ complexity of AE waveforms in identifying the damage modes in Carbon Fibre Reinforced Polymer (CFRP) composites was studied [12]. Nonetheless, the amount of research dedicated to LZ complexity in AE applications is very limited. 

In this research work, AE waveforms are recorded from the mechanical testing of CFRP composites. The LZ complexity indices of the acoustic waves are calculated and are used to characterize the mechanical properties of the CFRP composites under loading. For this k-means++ algorithm, a data clustering algorithm is used. A Convolutional Neural Network (CNN) framework was built to validate the characterization strategy proposed using LZ complexity. Time–frequency spectrograms of the AE waveforms generated using Continuous Wavelet Transform (CWT) were used for training and testing the CNN. Detailed information about the methodology used in this research work is explained in the subsequent sections. The data clustering, CWT, calculation of LZ complexity indices, and training and testing of CNN are executed in the programming module of MATLAB R2022b. 

## 2. Materials and Testing Methods

### 2.1. Materials

The CFRP composites used in this study were prepared by a vacuum bag molding process using laminate prepregs. The prepregs were prepared by hot melt method for resin impregnation method. Epoxy resin, Toray 2510, was impregnated through carbon fibres stitched in plain weave fabric configuration. The average fibre density in warp and fill directions are the same (1.8 g/cm^3^). The fibres are high strength carbon fibres of tensile strength 4900 MPa and elastic modulus in tension of 240 GPa. The average resin percentage in the prepreg is 41.5%. The prepared prepregs have a nominal thickness of 0.218 mm. 

A total of eight plies were used for preparing the composite slabs by the vacuum bag moulding process. The pressure inside the vacuum bag is kept at 22 in. of Hg and the composites are cured at a temperature of 132 ℃. The curing time of the composite slabs is 120–150 min. 

Five tensile test specimens are cut from the composite slabs longitudinally along the warp direction of the fibres. The dimensions of the specimens are as per the ASTM D3039 standard [13]. The test specimens are constant rectangular cross-sections with an average width of 25.01 ± 0.16 mm and thickness of 1.80±0.03 mm. The width and thickness were measured for each specimen in five different positions and their average values are reported above. The total length of the specimens is 200 mm. Leaving approximately 80 mm of useful section, the end tabs were glued to the tensile test specimens using Hysol EA9628 adhesive. 

### 2.2. Test Methods and Data Acquisition 

Tensile tests were carried out in an INSTRON servo-hydraulic test machine (Norwood, Massachusettsas, USA) as per the ASTM D3039 standards [13]. A couple of HBM uniaxial strain gauges (with R = 350 Ω) were bonded to the midspan of the tensile specimens, one along the longitudinal direction and another along the transverse direction. The longitudinal and the transverse strain measured during the tensile loading were acquired using a multichannel Quantum X data acquisition system. The load cell and the crosshead displacement sensors are also attached to this multichannel data acquisition system. 

For recording the acoustic emission signals generated during the tensile test, a broadband piezoelectric transducer Pico sensor (Physical Acoustics Corporation, Mistras Group, NJ, USA) of operating frequency 250 kHz–750 kHz was used. The sensor was coupled to the surface of the specimens. A thin layer of silicone grease was smeared between the sensor and the specimen surface to compensate the presence of air gap. To acquire the useful signal from the damage progression in the test specimens, the signals only above the detection threshold of 35 dB were acquired. These signals were then preamplified by 40 dB using a 2/4/6 switch selectable gain single ended and differential pre-amplifier (Physical Acoustics, MISTRAS Group, NJ, USA). Furthermore, to improve the signal-to-noise ratio, the acquired signals were filtered through low band-pass and high band-pass filters of 100 kHz and 1 MHz. Signal waveforms of length 1K were recorded at a sampling frequency of 2 MHz.

The tests were carried out at an elevated temperature of 120 ℃. The test specimens were placed inside an environmental chamber, where the temperature was ramped up to 120 ℃. The sensors used in this study can be operated up to 175 ℃, therefore all data were recorded without any loss. This study focuses on the mechanical characterization using the information–theoretics parameter of the AE signal. Therefore, the influence of the elevated temperature on the tensile properties was not considered during this study. Besides, there is no evidence in the literature to indicate that the acoustic wave propagation is affected by the test temperature, considering the temperature is well below the glass transition state of the composites (which is 141 ℃). Readers who are interested in the effect of test temperature in the mechanical properties of the CFRP composites are directed to the authors’ previous research work and other relevant research works [14,15].

## 3. Proposed Methodology 

The four-step methodology used in this research work is explained in detail in this section. First, the relationship between commonly used AE descriptors, peak amplitude and number of counts, and the information–theoretic parameter LZ complexity was studied. The peak amplitude is the voltage peak of the recorded signal, measured in decibels (dB). The number of counts is the total number of instances the voltage peak in the recorded signal crosses the detection threshold of 35 dB. Details about LZ complexity are explained separately in Section 3.1.

Peak amplitude and counts are more than often used in analysing the AE signals; however, their reliabilities are debated on more than one occasion. A consensus among the AE research community is that utilizing more than one AE parameter often provides better results [16,17,18,19,20]. This is the reason for trying to establish a relationship between the peak amplitude, counts, and LZ complexities of the AE signal, so that the latter can be validated as a useful AE descriptor.

For this purpose, a data clustering technique, k-means++ algorithm, was used for classifying the AE signals into predefined number of classes based on their peak amplitude and counts. After that, the LZ complexities of the AE signal in each class were calculated. This yields the relationship between peak amplitude, counts, and LZ complexities of the AE signals. 

Second, to characterize the mechanical properties of the CFRP composites, the LZ complexities of the classified AE signals and the tensile test data were compared. Through this, the LZ complexities of the AE signal at different stress levels, strain levels, and loading stages of the tensile tests were evaluated.

Third, to validate this characterization, the classified AE signals were analyzed in their time–frequency domain using Continuous Wavelet Transform (CWT). Provided that the classification strategy is satisfactory, the time–frequency characteristics of the AE signals classified in each stage must be similar.

Finally, this similarity was verified by building a CNN and training it with the CWT spectrograms of the AE signals from each class. The validating efficiency of the CNN in classifying the spectrograms of each class is approximately the efficiency of the classification strategy proposed using peak amplitude, counts, and LZ complexity.

**Figure 1:**
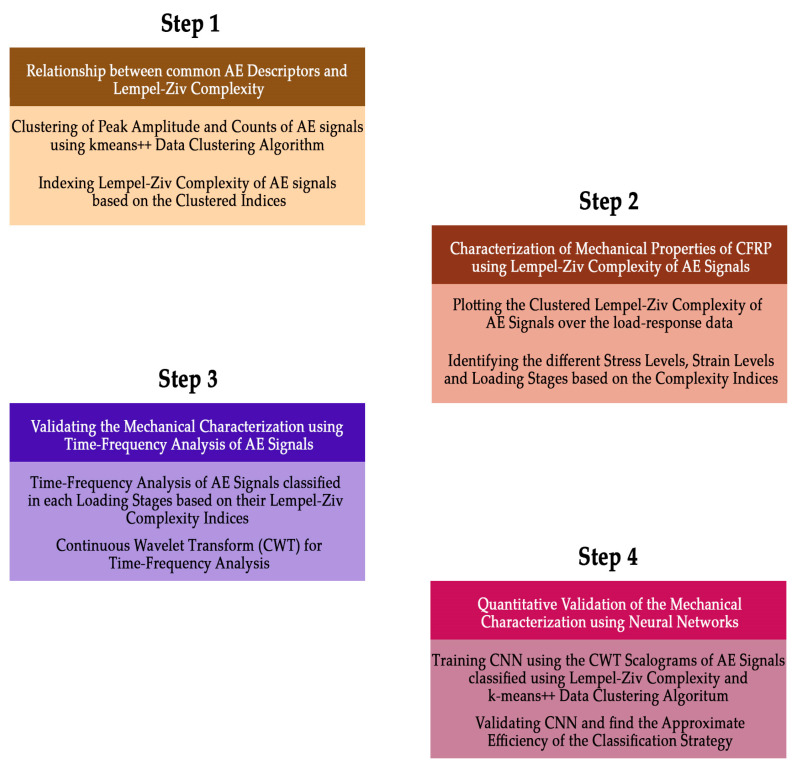
Four-Step Classification Methodology for Mechanical Characterization of CFRP Composites using Acoustic Emission Signal Data.

This four-step methodology was proposed to validate LZ complexity as an efficient AE descriptor in studying the mechanical behaviour of the CFRP composites under tensile loading. A schematic of this four-step methodology is presented in Figure 1.

Brief details about the LZ complexity, k-means++ data clustering algorithm, and signal processing using CWT and CNN frameworks are explained in the subsequent subsections. Since all these procedures are well-documented in the literature and extensively used in several applications, only a brief description is presented in this research work.

### 3.1. LZ Complexity 

Every time-series data may have random looking sequences. In time-series data of the waveforms, these sequences may not be random. Particularly, the presence of white noise, periodic noise, or harmonics results in the repetition of these sequences [11,21]. The complexity is a measure of the extent to which the given sequence resembles a random one. 

The identification of these randomly appearing sequences can be made possible by converting the time-series data of the waveform into a series of finite elements of symbols. Traditionally, for calculating the complexity, the time-series data are converted into a series of binary sequence. 

LZ complexity was proposed by Lempel and Ziv [11], which is related to the number of steps in a self-delimiting production process by which a given sequence is presumed to be generated. 

In this research work, the recorded acoustic signal was converted into its analytical form using Hilbert Transform. The process is explained in detail in the authors’ previous research work [12]. If the time-series data of the AE signal waveform are expressed as S, then the Hilbert transform of the signal can be expressed as H=abs [hil(S)], where H will be in the form $\left\{h_{1},\;h_{2},h_{3},\ldots ,\;h_{n}\right\}$. $h_{i}$ is the absolute component of the analytical signal data and n is the length of the signal. The analytical form of the signal data is converted into its binary sequence by the condition explained in Equation 1.

(1) $$b=\{\begin{array}{c} 0,\;\;if\;h_{i}<t_{h}\\ 1,\;otherwise\; \end{array},$$

The threshold for the conversion $t_{h}$ is selected as the median of H. Now, from this finite binary sequence, the LZ complexity index of the signal waveform is calculated. The detailed procedure about the LZ complexity with an example can be found in the authors’ previous research work [12]. The procedure was repeated to calculate the LZ complexities of all the waveforms recorded during the tensile test of the CFRP specimens. 

### 3.2. k-means++ Data Clustering Algorithm 

As mentioned earlier, the AE data recorded during the tensile tests are classified into a predefined number of clusters based on their peak amplitude and number of counts. For this purpose, k-means++ algorithm is used. It is a data partitioning algorithm which assigns the two-dimensional AE data (of peak amplitude and counts) into k number of clusters. The optimal number of clusters to which the data must be classified is obtained by the Davies Bouldin Index (DBI). 

The k-means++ algorithm classifies the data by computing the distance between each datapoint and centroids. Initially, the k number of centroids are chosen, which is followed by computing the distance between each datapoint and the cluster. In this work, the distance metrics used is Euclidean distance. The datapoints were assigned to the cluster with the closest centroid. The average value of the distance between the centroid-to-datapoints in each cluster was calculated to obtain new centroid locations. The process of calculating new centroid positions and assigning of datapoints to each cluster is repeated until the cluster assignments do not change or the number of iterations for the process repetition is reached. 

The initialization of the centroids, although a heuristic process, ultimately reduces the running time of the algorithm. The details of the step-by-step procedure of centroid initiation and the cluster assignments are well-documented and can be found elsewhere [17,20]. In fact, the k-means++ data clustering have been employed in a significant number of research works for clustering AE data. 

Traditionally, the clustered AE data are directly assigned to the AE signals generated from different damage modes of CFRP composites such as fibre breakage, delamination, matrix cracking, or debonding. In fact, in the authors’ previous research work, this procedure was used for similar applications [22,23]. In this research work, however, the clustered data are used for characterizing the mechanical properties of the composites, which will be explained in Section 4. 

### 3.3. Continuous Wavelet Transform 

The AE data classified into different clusters are analyzed in their time–frequency domain using Continuous Wavelet Transform (CWT). A wavelet transform is a method of decomposing a signal into a set of elementary waveforms [24]. Fourier transform uses sine waves to decompose the signal, whereas wavelet transforms use mother wavelets. Wavelet transforms are preferable for analysing AE signals because the characteristic natures of the AE signals are transient, unstable, or often decaying sinusoidal. Therefore, if the signal is decomposed using sine waves, the wavelet coefficients of the decomposed elementary waveforms do not yield good results. Typically, Morlet wavelet or analytical Morlet wavelet are used in CWT of AE signals [25,26]. In this study, however, the bump wavelets are used, which provides higher frequency localization. The graphical representation of the wavelet coefficients of CWT, which also provides the spectral energy of the coefficients, is termed as spectrograms. These spectrograms are used for time–frequency analysis of the AE signals in this study. In addition to this, the scalograms of these waveforms are used as the input for training the CNN. Other details about different types of wavelets, the CWT procedure are explained in detail in textbooks [24]. 

### 3.4. Convolutional Neural Network 

Convolutional Neural Networks are introduced primarily for the machine vision applications to detect patterns or objects. They are also extensively used in computer vision. The pattern detection capability of CNNs made them formidable in identifying the patterns in waveform signal data. Signal time in their time-series representations can be directly fed into a pre-trained CNN to identify their category. In recent years, the detection capabilities of CNN have been improved by varying the architecture of the neural network frameworks or by changing the input forms into scalograms of Discrete Wavelet Transform (DWT), Mel scale, Short-Time Fourier Transforms (STFT), and so on [27,28,29]. In fact, CNN are used to identify the localized time–frequency patterns of AE signals generated from various types of materials and processes [30,31,32]. 

A typical CNN architecture consists of an input layer, which is connected to various hidden layer, and ends with an output layer. The hidden components are convolutional layer, pooling layer, and classifier layers. Each convolutional layer contains varying sizes of kernels, which convolves the input to produce a feature map. The outputs of the convolutional layers are downsampled by a pooling layer. A pooling layer is always connected to the convolutional output. Based on the requirements, the pooling layer may calculate the maximum or average value of the convolutional output. The output of the pooling layer is activated by an activation function. ReLu (rectified linear function) or a tanh function are commonly used for activation. Prior to the classifier layer, Softmax activation function is used, which outputs the vector values in the range of (0, 1). The values represent the probability of the input data belonging to one of the possible categories. 

**Figure 2:**
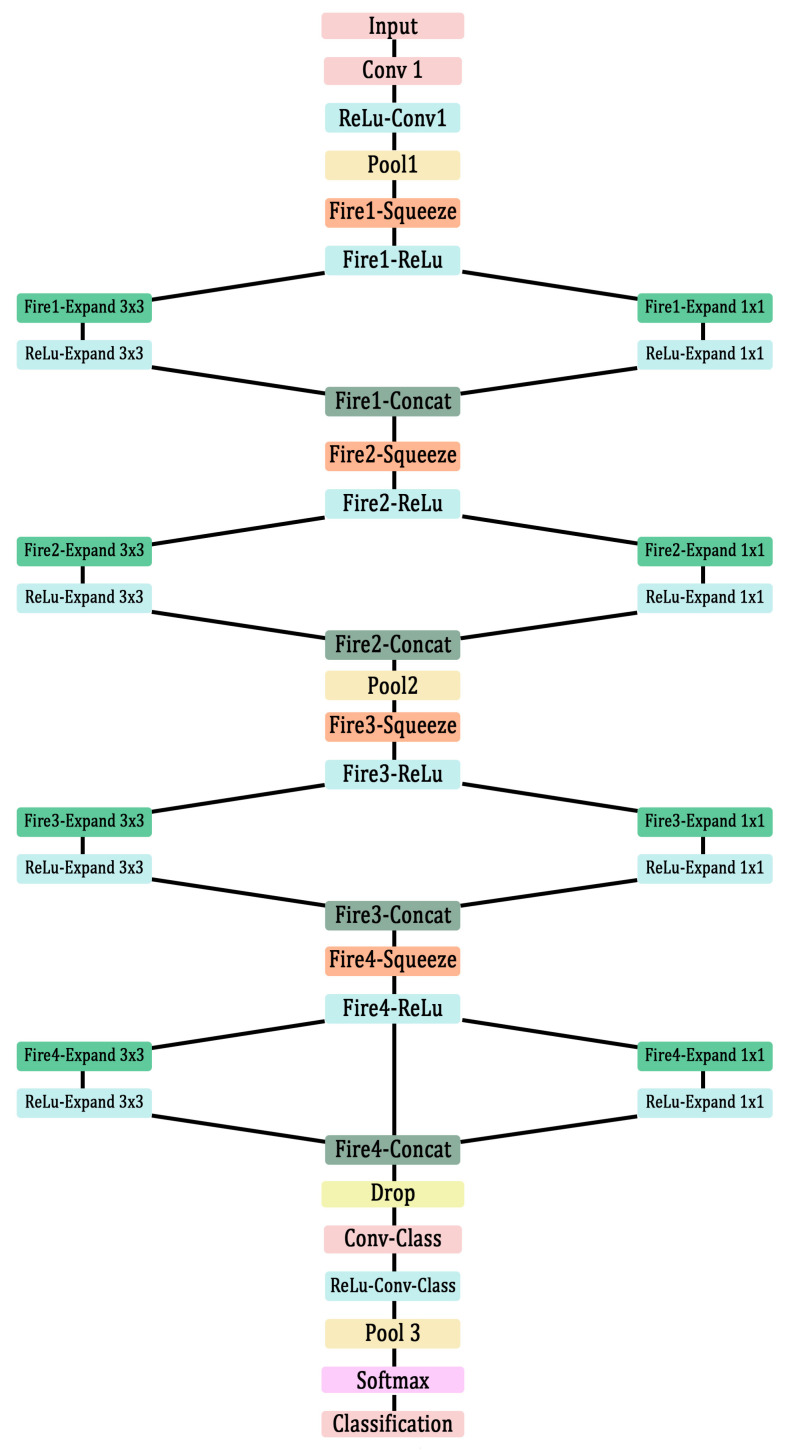
Architecture of SqueezeNet-based CNN.

In this research work, a CNN based on SqueezeNet, which is available in the Deep Network Designer toolbox in MATLAB, was used [32,33]. Details about the SqueezeNet can be found elsewhere. In this section, the architecture of the SqueezeNet used in this work and their details are elaborated in Figure 2 and Table 1. 

The general architecture of SqueezeNet in MATLAB is used for identifying 3D objects in 2D inputs, which is not necessary for analysing the scalograms of the AE signals. Therefore, some of the deeper layers with large number of filters were removed, and dropout layers were added to avoid the overfitting of data. This network was used in the authors’ previous research work for identifying damage modes in AlSi10Mg tensile test specimens with success [32]. Therefore, the same architecture is considered for this study.

The network was trained based on the Stochastic Gradient Descent (SGD) algorithm. The parameters used for training the network are briefed in Table 2. 

## 4. Results and Discussions

As mentioned in Section 3, the results in this research work are presented and discussed in four steps. To summarize, the results are obtained by analysing the AE data and the mechanical data obtained from the tensile tests of CFRP composite test specimens. Five different specimens are tested, which are named as T-001 through T-005.

### 4.1. AE Data Clustered Based on Peak Amplitude and Counts 

First, the distribution of the AE signals, based on their descriptors counts and peak amplitude, was classified using k-means++ algorithm. Similar works have been reported by researchers where different AE descriptors, such as counts, risetime, peak amplitude, were classified using a data clustering algorithm [34,35,36]. DBI was used for identifying the optimal number of clusters to which the AE data can be classified. This index was used successfully in evaluating the cluster of AE data for damage analysis by many reviewers [19]. 

**Figure 3:**
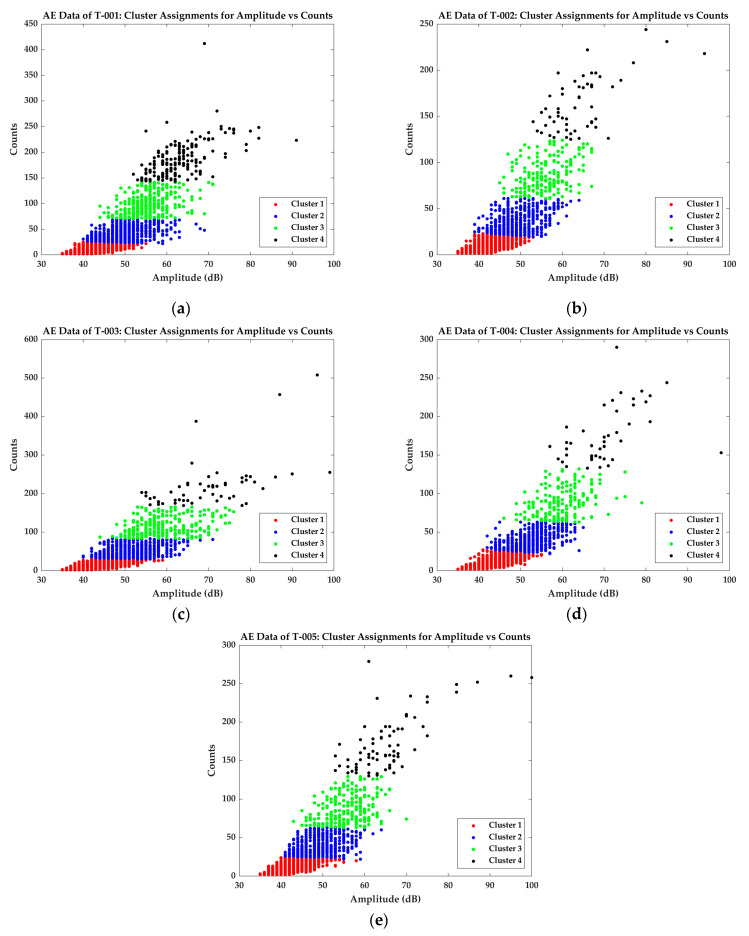
Clustered data based on amplitude and counts of the AE signals recorded from the tensile test of specimens (**a**) T-001; (**b**) T-002; (**c**) T-003; (**d**) T-004; (**e**) T-005.

DBI is calculated for cluster values k=2−6. The cluster number k is associated with the minimum value of DBI is the optimal number. Based on the DBI calculated for AE data from all the test specimens, it is appropriate to classify the data into four clusters based on their peak amplitude and counts (Figure 3). 

The data clustered using k-means++ algorithm show very similar patterns, which shows the good repeatability of AE data acquisition. The similarities in the clustered AE data are summarized in Table 3. Cluster 1 of the AE signals generally have amplitude between 35 dB and 55 dB and counts less than 25. Signals from Cluster 2 have amplitude between 40 dB and 60 dB and counts between 26 and 55. Signals from specimens T-003 and T-004 have some signals with amplitude close to 65 dB. Considering the global average of the AE signals in this category, they can be considered as outliers. Signals in Cluster 3 have an amplitude between 45 dB and 65 dB (ignoring the outliers) and have counts up to 150 (170 in case of specimen T-003). Cluster 4 has high amplitude signals with higher counts value. The signals in these cluster have an amplitude greater than 50 dB and counts more than 150. 

Many researchers classify the AE signals based on two AE descriptors using data clustering algorithms and associate them directly to different damage modes. For example, in many research works, AE signals from Cluster 1 are associated with the signals from matrix cracking events [16,18,20]. Matrix cracking is considered to generate signals with lower amplitude values. This method of damage classification is flawed, as the data clustering depends only on the distribution of the AE data. As explained in Section 3.2, k-means++ algorithm assigns the data to each cluster based on the average distance between them. Therefore, associating the clustered data without further analysis is meaningless. The clustering data in Figure 3 only shows the repeatability of data acquisition between the five different tests. In that case, how the relationship between these descriptors and their complexity indices can be established? The complexities of the AE signals in each of these categories are calculated and are plotted over the load response curve of the tensile tests. The results follow in the next section. 

### 4.2. Relationship between the Acoustic Emission Descriptors, LZ Complexity, and Tensile Test Results

**Figure 4:**
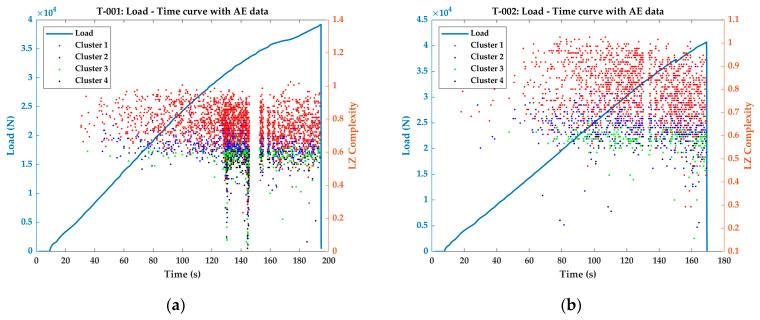

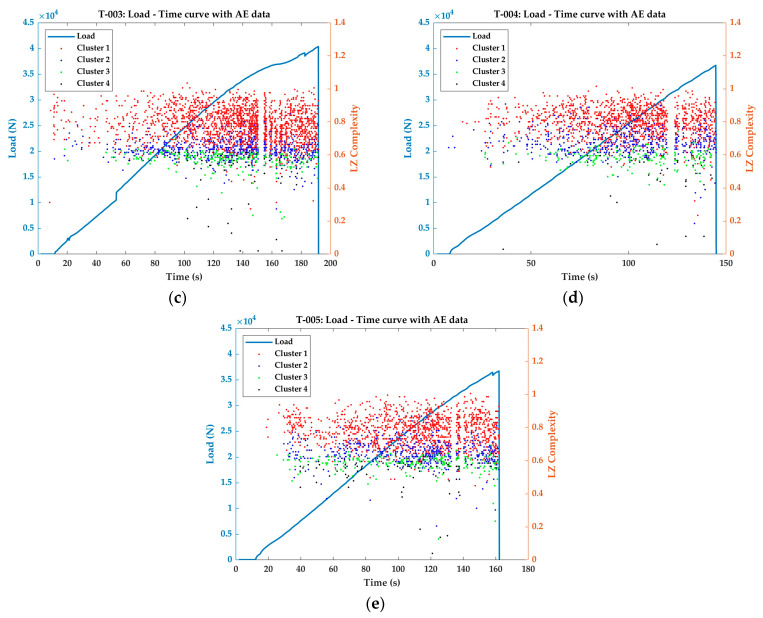
Classified LZ complexities of the AE signals plotted over the load–time curve of the tensile tests (**a**) T-001; (**b**) T-002; (**c**) T-003; (**d**) T-004; (**e**) T-005.

The LZ complexities of the classified AE signals from the tensile tests are plotted over the load response curves, which is presented in Figure 4. 

During the early stages of loading, the amount of the AE signal generated and their general distribution density is very low in all the cases. This means that during the elastic phase of the material, AE signal generation is seldom observed in most cases. However, this is not the case in these plain weave fabric CFRP specimens (refer to the AE distribution during early stages of loading in Figure 4a through Figure 4e). In plain weave fabric CFRP specimens, when the load is applied along the fibres in the warp direction, the majority of the load is carried by the fibres. The resin matrix present between the warp and fill directions are squeezed, and this local compression induces micro-failures such as matrix cracking [37,38,39]. These micro-cracks do not significantly affect the load-carrying capabilities of the tensile specimens, provided there are no resin rich areas, and the density of the resin is uniform throughout the specimen [37,38,39]. Therefore, the elastic phase extends for a longer period and there is little to no plastic phase before the final failure. The stress–strain (longitudinal) results of the tensile tests are presented in Appendix A.

Although these micro-failures do not affect the longitudinal strain evolution in the specimens, the released elastic energy during these failures propagates as acoustic waves of lower amplitude. Consequently, Cluster 1 AE signals were observed throughout the loading phase of the test specimens in Figure 4a through to e. It can be observed from Figure 4 that the complexity indices of these Cluster 1 AE signals are between 0.6 and 1. This shows that the AE signals generated from the micro-failures of matrix cracking belong to Cluster 1 and have a complexity index between 0.6 and 1. 

Similar to Cluster 1 AE signals, Cluster 3 AE signals are also distributed throughout the loading phase of the tests. However, unlike Cluster 1 signals, Cluster 3 signals have LZ complexity indices mostly between 0.4 and 0.65 (generally less than 0.65).

The interesting relationship between the tensile test results and the LZ complexity indices of the AE signals is apparent while seeing Cluster 2 and Cluster 4 AE signals. Cluster 4 AE signals have higher amplitude (>50 dB) and large number of counts (>150) and the complexity indices generally below 0.6 starts to appear only after a certain stage of testing. The stage where Cluster 4 region starts to occur is named as Region of Interest (ROI) for the rest of this study. The mechanical properties of the test specimens at the ROI are extracted and reported in Table 4.

Interestingly, the occurrence of Cluster 4 signals contains significant information about the tensile test results. From Table 4, it can be observed that the transversal strain at the ROI has an average value of −459.84 µε with a very small standard deviation of 7.35. Second, despite the transversal strain at ROI returning a very similar value, their longitudinal strain values, the occurrence of Cluster 4 signals, and their stress values strongly influence the tensile test results. In specimens T-001, T-002, and T-003, the Cluster 4 signals occur at later stages of loading (129.8 s, 97.0 s, and 112.0 s, respectively). The stresses at ROI in these specimens also have smaller variance. The Ultimate Tensile Strength (UTS) of these three specimens has an average of 902 ± 14 MPa. 

In specimens T-004 and T-005, however, the Cluster 4 signals occurs quite early during the loading phase (79.0 s and 66.9 s, respectively) and at a longitudinal strain of 7013.49 µε and 6021.14 µε, respectively, which are less compared to the other three specimens. Similarly, these stress states at ROI of the specimens T-004 and T-005, respectively, are 436 MPa and 328 MPa. The UTS of these specimens are 819 MPa and 816 MPa, which are very low compared to the average value of 902 MPa of the other three specimens. 

The accumulation of micro-failures in composites leads to a final failure, which probably could have been initiated at ROI. This results in the generation of Cluster 4 signals at this specific region. Catastrophic failures in composite specimens, where it is due to the through-thickness crack growth, longitudinal splitting, or fiber debonding, depending on the configuration of the specimen [38,39], often generate higher amplitude signals near failure [10,33,34]. Considering that Cluster 4 signals have higher amplitude, these signals are associated with the final failure. Therefore, the earlier occurrence of these signals in Specimens T-004 and T-005 results in lower UTS in these specimens compared to the T-001, T-002, and T-003. 

Finally, looking at the Cluster 2 signals, it may seem that they occur generally throughout the loading history and have complexity indices between 0.5 and 0.7. However, after the ROI, Cluster 2 signals with LZ complexities greater than 0.7 start to appear. There is a clear absence of Cluster 2 signals with LZ complexities greater than 0.7 prior to the ROI, despite sharing the same features in terms of amplitude and counts. 

The results presented in this section show the relationship between the three AE descriptors and their relationship with the mechanical properties of the tensile specimens. More importantly, LZ complexity indices of the AE signal prove to be an additional effective tool for associating the AE signals to the mechanical characteristics of the test specimens. Unlike the previous studies where the clustered data are used to identify the failure modes [22,23,34,36], the discussions are not focussed on identifying the failure modes. The results describe that the AE signals generated from the tensile tests are classified into four clusters. Among the four clusters, Cluster 2 and Cluster 4 and their LZ complexity indices are quite useful in identifying the critical regions of failure and the mechanical properties associated with them. 

### 4.3. Validation of the Clustered Results Using Continuous Wavelet Transform

**Figure 5:**
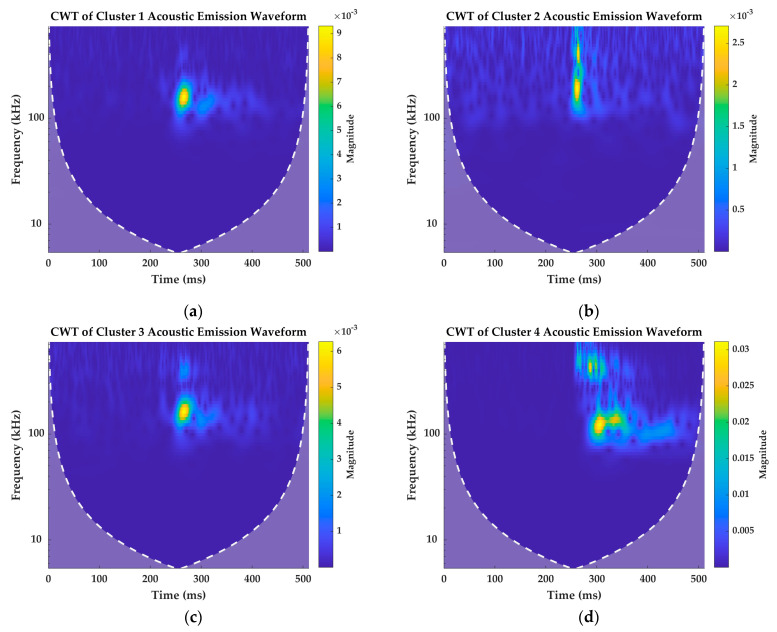
CWT spectrograms of AE signals taken randomly from (**a**) Cluster 1; (**b**) Cluster 2; (**c**) Cluster 3; (**d**) Cluster 4.

As mentioned in the earlier section, simple classification of signals based on two different parameters are affected by the data distribution. This is the reason why these results are associated with a third parameter, LZ complexity, and validated by them. Each of the clustered data can be classified based on their complexity indices. Nonetheless, to validate these results qualitatively, signals from each of these clusters are taken and analyzed in their time–frequency domain. Four signals taken from the four clusters are analysed using Continuous Wavelet Transform (CWT) and their spectrograms are presented in Figure 5. However, the CWT is performed for all the signals generated during the test. A sample of four signals per each cluster is presented in Appendix B. 

Cluster 1 AE signal has low frequency and lower amplitude localized for a shorter duration. The spectrogram of the AE signal in Figure 5a shows that the magnitude of the spectral density is maximum around 150 kHz frequency band and has a value of $9e^{-3}$. Cluster 2 AE signals, however, have a very transient signal with the maximum frequency localized in two frequency bands: one around 150 kHz and another at a higher frequency band. The maximum magnitude of the spectral density of this signal in Figure 5b is $2.5e^{-3}$. 

The spectrogram of Cluster 3 signal in Figure 5c is quite similar to the spectrogram of the signal in Cluster 1. However, there is a presence of second frequency band, but with a lower spectral density at higher frequency band, which is absent in Cluster 1. The maximum magnitude of the spectral density at 150 kHz frequency band is around $6e^{-3}$ and at the second frequency band is $2.5e^{-3}$. Cluster 4 signal in Figure 5d has larger reverberations compared to the spectrograms of other signals. Since these signals are from higher amplitude clusters, the magnitude of the spectral density is also quite high, which is around $3e^{-2}$. 

The distinct time–frequency features of the signals presented in Figure 5 and the other randomly selected signals from each cluster presented in Appendix B validates the classification results discussed in Section 4.1 and Section 4.2. The spectrograms qualitatively show that the signals are classified based on their peak amplitude, counts, and LZ complexity indices that show distinct features. 

### 4.4. Quantitative Validation of the Clustered Results Using Convolutional Neural Network Framework

Figure 5 and the spectrograms in Appendix B show the similarity in AE signals grouped in four clusters. However, these results show only a part of the entire data. The total number of signals generated during the tensile tests of five specimens is much higher to discuss them with limited number of signals. From the five tensile tests, the total number of AE signals generated was 13370. Among them, 8798 signals are from Cluster 1, 2819 from Cluster 2, and 1169 and 404 are from Clusters 3 and 4, respectively. The percentage of AE signals in each cluster from each individual test is presented in Table 5. 

If the classification proposed and explained in Section 4.1 and Section 4.2 holds true, then the percentage of similarity from AE signals from each cluster must be very high. For this purpose, the CWT scalograms of the AE signals from each cluster are used for training the CNN and the test is run to validate the results. CNN is capable of identifying images based on their similarities. If the scalograms of the AE signal from each cluster is similar, then CNN must be able to associate them to each cluster with 100% efficiency. 

However, CNN can be trained more efficiently if the number of input data is the same. For this, randomly generated white noise is added to the signals from Clusters 2, 3, and 4, and is augmented. The final augmented dataset has 8000 signals in each cluster, a total of 32,000 signals. Overall, 6000 signals from each cluster were used for training the CNN and 2000 signals were used for validating. The validating efficiency of the CNN can approximately be considered as the efficiency of the classification procedure proposed in this research work. 

**Figure 6:**
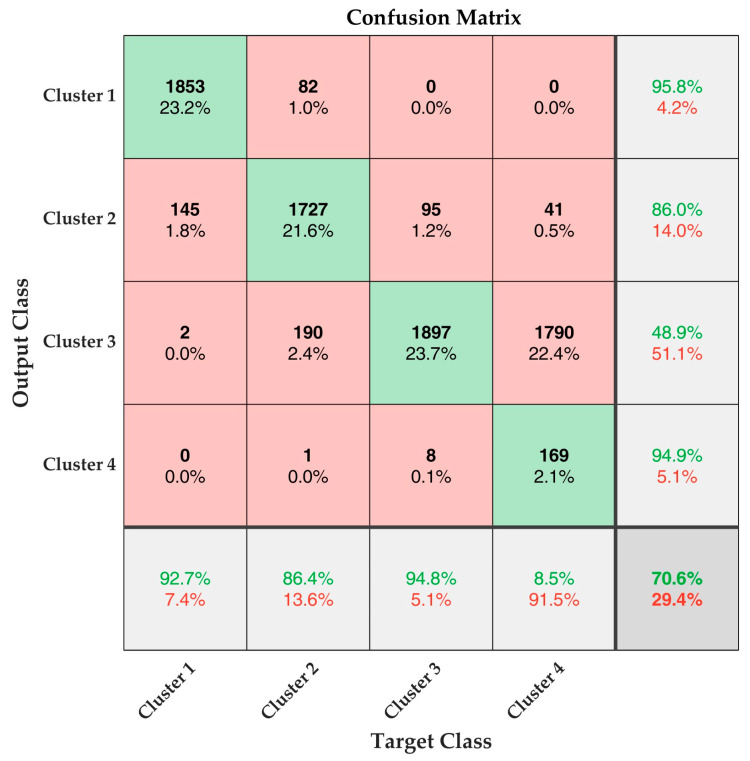
Classification Efficiency of the SqueezeNet-based CNN represented as Confusion Matrix.

The CNN constructed using the architecture explained in Figure 2 and Table 1 were trained using the training parameters reported in Table 2. The validation dataset of 2000 signals were then classified using this pretrained network. The results are presented in Figure 6. 

The confusion matrix results presented in Figure 6 shows that the CNN classified the signals in Cluster 1, Cluster 2, and Cluster 3 at an efficiency of 92.7%, 86.4%, and 94.8%, respectively. However, the signals in Cluster 4 are classified only at a very low efficiency of 8.5%. This reduces the overall classification efficiency of the CNN to 70.6%. However, it can be noted that majority of the signals in Cluster 4 are misidentified as Cluster 3 by the CNN. It must be noted that the percentage of signals in Cluster 4 is very low (refer Table 5), and the total number of signals were merely 404. These signals were augmented to 8000 for training and testing the CNN. The augmentation possibly could have resulted in this poor efficiency. The classification efficiency of CNN can easily be rectified by changing the type of input or upgrading the training procedure using k-fold cross-validation. In the authors’ previous research work, k-fold cross-validation was used to achieve 100% classification efficiency of CNN [32]. 

However, the goal of this research work is not to achieve 100% efficiency but to retrieve the true classification efficiency of the clustered AE data. In this regard, it is safe to say that the classification strategy proposed in this research work functions at an efficiency of more than 85% for Clusters 1, 2, and 3. The low efficiency of Cluster 4 could not be associated with the method used but was rather due to the data augmentation. 

This shows that the classification strategy of AE signals proposed using peak amplitude, counts and LZ complexity index is very efficient. Particularly, this validates the utilization of an information–theoretics parameter, which is independent of the sensor characteristics or data acquisition characteristics, as an efficient parameter for AE data analysis for mechanical characterization. 

## 5. Conclusions

In this research work, five tensile test specimens of plain weave fabric CFRP specimens were tested, and their mechanical properties were analyzed using the AE signals generated during the loading. A four-step methodology was proposed for analyzing the AE signals associated with the tensile tests.

- The characteristics of the AE signals were analysed in terms of their amplitude, counts, and LZ complexity indices. The AE signals with amplitudes above 50 dB, counts greater than 150, and LZ complexity indices below 0.6 initiate at a region of critical failure (ROI). The transversal strains at ROI of the test specimens exhibit a very similar value of −459.84 µε with a very small standard deviation of 7.35. The longitudinal strains and the tensile stresses at ROI vary between specimens, which can be used to identify the specimen with poor strength or the specimen, which is susceptible to earlier damage. Thus, the critical ROI identified by the AE signals are capable of identifying the major failure occurrence of the test specimens.
- The AE signals from different clusters are validated for their similarity using CWT spectrograms.
- Finally, a quantitative similarity is calculated by using CNN. The results show that the classification procedure is more than 85% efficient for classifying the AE data for signals in Cluster 1, Cluster 2, and Cluster 3.

Unfortunately, the efficiency of identifying Cluster 4 is very low, which limits the practical application of this approach at this moment. Nonetheless, a deeper investigation of the proposed approach will help in implanting this technique in the application of identifying failure initiation and characterizing damage progression in CFRP composites. One of the advantages of this proposed approach is the utilization of LZ complexity, which is independent of the choice of the sensor. The future scope of this work is to validate this approach on different configurations of FRP materials and their damage progression in various loading conditions. 

## Figures and Tables

**Table 1:** Details about the layers in the SqueezeNet-based CNN used in this study.

Fire Module n	Layer Name	Layer Description
Squeeze	Fire*n*-Squeeze	Number of Filters: X Filter Size: 1 × 1 Stride: 2 × 2 ReLU activation
Expand	Fire*n*-Expand 1 × 1	Number of Filters: X Filter Size: 1 × 1 Stride: 2 × 2 ReLU activation
	Fire*n*-Expand 3 × 3	Number of Filters: X Filter Size: 1 × 1 Stride: 2 × 2 ReLU activation
Concatenation	Fire*n*-Concat	

**Table 2:** Parameters for training the SqueezeNet-based CNN.

Training Parameters	
**Initial Learning Rate**	0.001
Learning Schedule	Piecewise
Drop Rate Factor	0.1
Drop Rate Period	8
Maximum Epochs	20
Minibatch Size	75

**Table 3:** Cluster details of the AE signals based on peak amplitude and counts.

Cluster	Peak Amplitude Range	Counts Range
Cluster 1	35–55 dB	<25
Cluster 2	40–60 dB	26–55
Cluster 3	45–65 dB	56–150
Cluster 4	>65 dB	>150

**Table 4:** Mechanical results at the region of interest (occurrence of Cluster 4 signals).

Specimen Name	UTS	Occurrence of Cluster 4	Stress at ROI	Strain at ROI	
		Time		Longitudinal	Transversal
	*Mpa*	*s*	*Mpa*	*µε*	*µε*
T-001	882	129.8	693	11225.94	-
T-002	912	97.0	543	9309.64	−468.18
T-003	912	112.0	624	6243.14 *	−450.31
T-004	819	79.0	436	7013.49	-
T-005	816	66.9	327	6021.14	−461.03

* Longitudinal strain was not recorded properly in Specimen T-003 after 500 Mpa.

**Table 5:** Percentage of AE signals in each cluster.

Specimen Name	Cluster 1	Cluster 2	Cluster 3	Cluster 4
T-001	69.36	17.75	4.81	8.08
T-002	68.09	21.39	8.23	2.30
T-003	69.16	20.63	8.40	1.81
T-004	65.61	23.34	9.04	2.01
T-005	60.96	24.94	10.66	3.44

**Figure A1:**
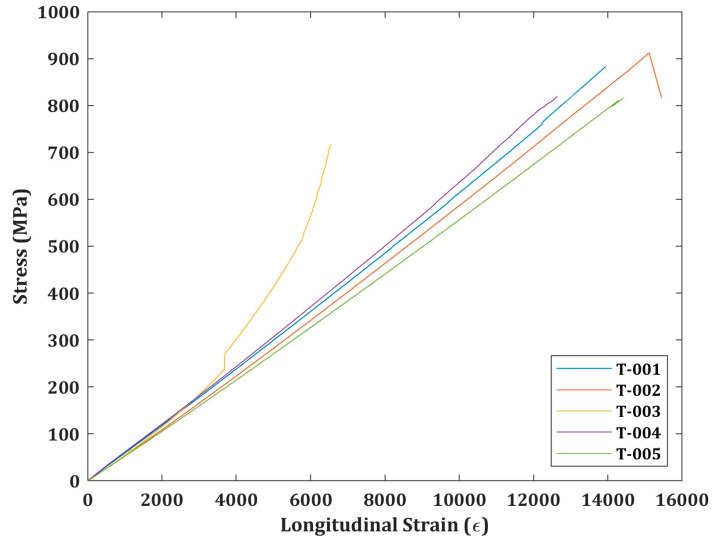
Stress–strain (longitudinal) curves of the tensile tests.

**Figure A2:**
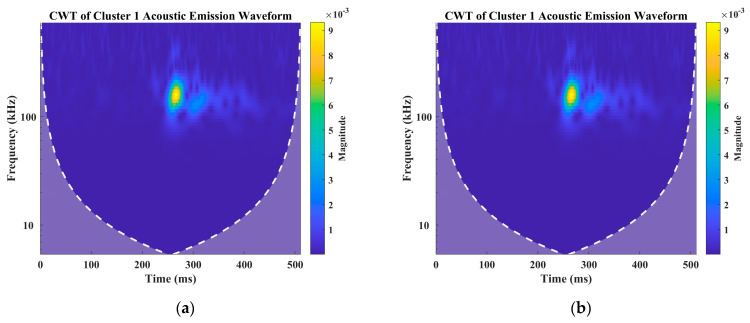

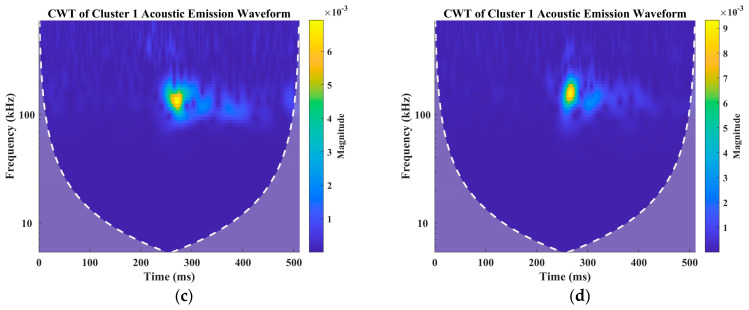
CWT spectrograms of four randomly selected AE signals (**a**) Signal 1; (**b**) Signal 2; (**c**) Signal 3; (**d**) Signal 4 from Cluster 1.

**Figure A3:**
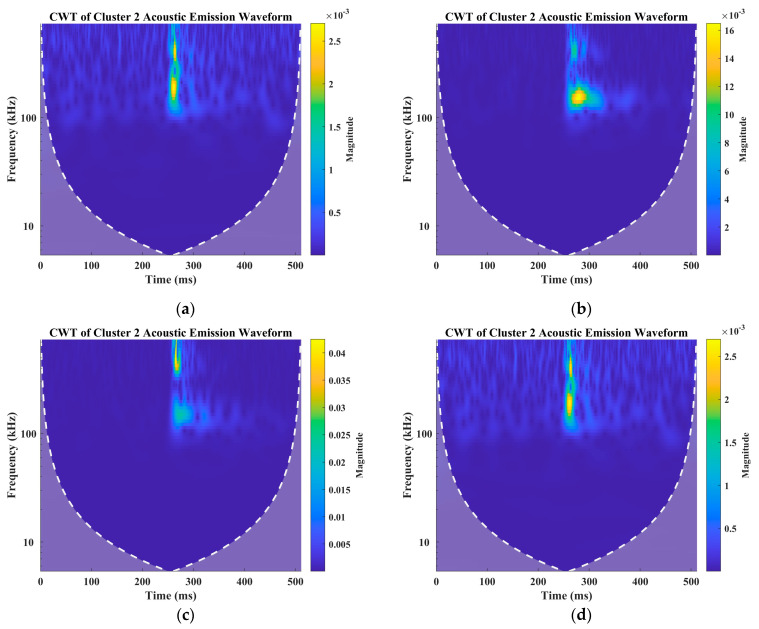
CWT spectrograms of four randomly selected AE signals (**a**) Signal 1; (**b**) Signal 2; (**c**) Signal 3; (**d**) Signal 4 from Cluster 2.

**Figure A4:**
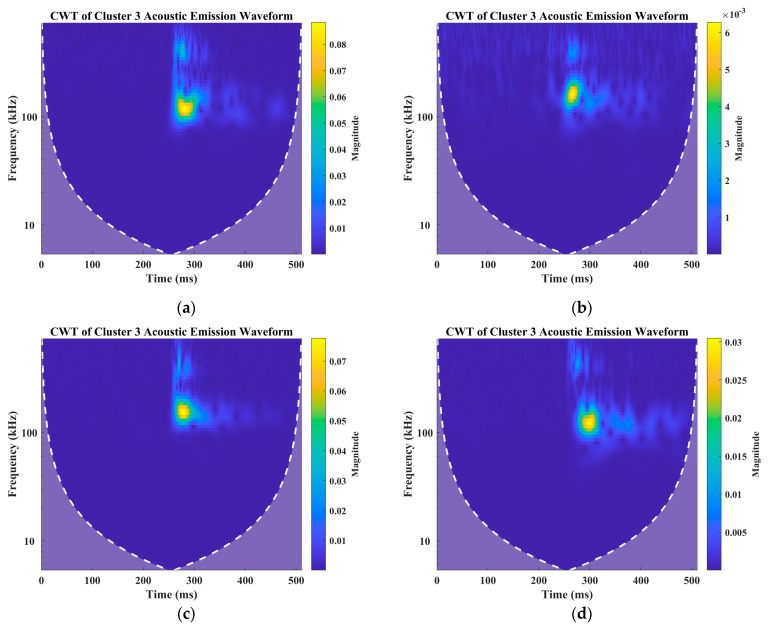
CWT spectrograms of four randomly selected AE signals (**a**) Signal 1; (**b**) Signal 2; (**c**) Signal 3; (**d**) Signal 4 from Cluster 3.

**Figure A5:**
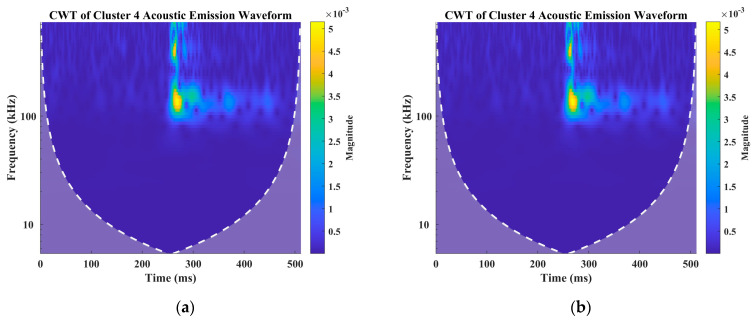

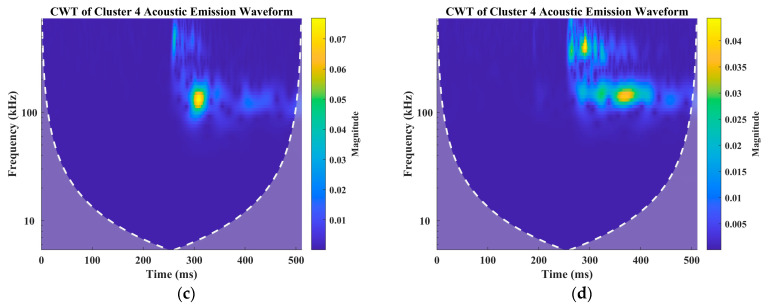
CWT spectrograms of four randomly selected AE signals (**a**) Signal 1; (**b**) Signal 2; (**c**) Signal 3; (**d**) Signal 4 from Cluster 4.

## Data Availability

The data presented in this research work are part of an extended research campaign and cannot be shared at this moment.

## Appendix A

The stress–strain (longitudinal) curves of the tensile tests are reported here. During the testing of specimen T-003, the outer ply failed in the midspan of the specimen, where the strain gauges are bonded. Therefore, the data collected is erroneous after the stress level of 500 MPa.

## Appendix B

CWT of four randomly selected signals from the four different clusters, Cluster 1, Cluster 2, Cluster 3, and Cluster 4 are presented in Figure A2, Figure A3, Figure A4, and Figure A5, respectively.

## References

[1] Hamstad M.A. (2000). Thirty Years of Advances and Some Remaining Challenges in the Application of Acoustic Emission to Composite Materials. Acoustic Emission beyond the Millennium.

[2] Barile C., Casavola C., Pappalettera G., Kannan V.P. (2020). Application of Different Acoustic Emission Descriptors in Damage Assessment of Fiber Reinforced Plastics: A Comprehensive Review. Eng. Fract. Mech..

[3] Hamstad M.A. (1986). A Review: Acoustic Emission, a Tool for Composite-Materials Studies. Exp. Mech..

[4] Liu P.F., Chu J.K., Liu Y.L., Zheng J.Y. (2012). A Study on the Failure Mechanisms of Carbon Fiber/Epoxy Composite Laminates Using Acoustic Emission. Mater. Des..

[5] Zhuang X., Yan X. (2006). Investigation of Damage Mechanisms in Self-Reinforced Polyethylene Composites by Acoustic Emission. Compos. Sci. Technol..

[6] Chandarana N., Sanchez D.M., Soutis C., Gresil M. (2017). Early Damage Detection in Composites during Fabrication and Mechanical Testing. Materials.

[7] Oskouei A.R., Zucchelli A., Ahmadi M., Minak G. (2011). An Integrated Approach Based on Acoustic Emission and Mechanical Information to Evaluate the Delamination Fracture Toughness at Mode I in Composite Laminate. Mater. Des..

[8] Baker C., Morscher G.N., Pujar V.V., Lemanski J.R. (2015). Transverse Cracking in Carbon Fiber Reinforced Polymer Composites: Modal Acoustic Emission and Peak Frequency Analysis. Compos. Sci. Technol..

[9] Fotouhi M., Pashmforoush F., Ahmadi M., Refahi Oskouei A. (2011). Monitoring the Initiation and Growth of Delamination in Composite Materials Using Acoustic Emission under Quasi-Static Three-Point Bending Test. J. Reinf. Plast. Compos..

[10] Aggelis D.G., Barkoula N.-M., Matikas T.E., Paipetis A.S. (2012). Acoustic Structural Health Monitoring of Composite Materials: Damage Identification and Evaluation in Cross Ply Laminates Using Acoustic Emission and Ultrasonics. Compos. Sci. Technol..

[11] Lempel A., Ziv J. (1976). On the Complexity of Finite Sequences. IEEE Trans. Inf. Theory.

[12] Barile C., Casavola C., Pappalettera G., Paramsamy Kannan V. (2022). Interpreting the Lempel–Ziv Complexity of Acoustic Emission Signals for Identifying Damage Modes in Composite Materials. Struct. Health Monit..

[13] ASTM A.M. (2017). ASTM D3039-Standard Test Method for Tensile Properties of Polymer Matrix Composite Materials.

[14] Barile C., Paramsamy Kannan V., del Core L., Casavola C. (2022). Tensile and Shear Behavior of Plain Weave Fabric Carbon Fiber Reinforced Polymer at Elevated Temperatures. Polym. Compos..

[15] Bazli M., Abolfazli M. (2020). Mechanical Properties of Fibre Reinforced Polymers under Elevated Temperatures: An Overview. Polymers.

[16] Roundi W., el Mahi A., el Gharad A., Rebiere J.-L. (2018). Acoustic Emission Monitoring of Damage Progression in Glass/Epoxy Composites during Static and Fatigue Tensile Tests. Appl. Acoust..

[17] Godin N., Huguet S., Gaertner R., Salmon L. (2004). Clustering of Acoustic Emission Signals Collected during Tensile Tests on Unidirectional Glass/Polyester Composite Using Supervised and Unsupervised Classifiers. Ndt E Int..

[18] Fotouhi M., Sadeghi S., Jalalvand M., Ahmadi M. (2017). Analysis of the Damage Mechanisms in Mixed-Mode Delamination of Laminated Composites Using Acoustic Emission Data Clustering. J. Thermoplast. Compos. Mater..

[19] Tang J., Soua S., Mares C., Gan T.-H. (2017). A Pattern Recognition Approach to Acoustic Emission Data Originating from Fatigue of Wind Turbine Blades. Sensors.

[20] Li L., Swolfs Y., Straumit I., Yan X., Lomov S.V. (2016). Cluster Analysis of Acoustic Emission Signals for 2D and 3D Woven Carbon Fiber/Epoxy Composites. J. Compos. Mater..

[21] Aboy M., Hornero R., Abásolo D., Álvarez D. (2006). Interpretation of the Lempel-Ziv Complexity Measure in the Context of Biomedical Signal Analysis. IEEE Trans. Biomed. Eng..

[22] Barile C., Casavola C., Pappalettera G., Vimalathithan P.K. (2019). Damage Characterization in Composite Materials Using Acoustic Emission Signal-Based and Parameter-Based Data. Compos. B Eng..

[23] Barile C., Casavola C., Pappalettera G., Vimalathithan P.K. (2020). Damage Propagation Analysis in the Single Lap Shear and Single Lap Shear-Riveted CFRP Joints by Acoustic Emission and Pattern Recognition Approach. Materials.

[24] Wickerhauser M.V. (1996). Adapted Wavelet Analysis: From Theory to Software.

[25] Lilly J.M. (2017). Element Analysis: A Wavelet-Based Method for Analysing Time-Localized Events in Noisy Time Series. Proc. R. Soc. A Math. Phys. Eng. Sci..

[26] Lilly J.M., Olhede S.C. (2008). Higher-Order Properties of Analytic Wavelets. IEEE Trans. Signal Process..

[27] Nasiri A., Bao J., Mccleeary D., Louis S.-Y.M., Huang X., Hu J. (2019). Online Damage Monitoring of SiC F-SiC m Composite Materials Using Acoustic Emission and Deep Learning. IEEE Access.

[28] Lin Y., Nie Z., Ma H. (2017). Structural Damage Detection with Automatic Feature-extraction through Deep Learning. Comput.-Aided Civ. Infrastruct. Eng..

[29] Sikdar S., Liu D., Kundu A. (2022). Acoustic Emission Data Based Deep Learning Approach for Classification and Detection of Damage-Sources in a Composite Panel. Compos. B Eng..

[30] Khan A., Ko D.-K., Lim S.C., Kim H.S. (2019). Structural Vibration-Based Classification and Prediction of Delamination in Smart Composite Laminates Using Deep Learning Neural Network. Compos. B Eng..

[31] Barile C., Casavola C., Pappalettera G., Kannan V.P. (2022). Damage Monitoring of Carbon Fibre Reinforced Polymer Composites Using Acoustic Emission Technique and Deep Learning. Compos. Struct..

[32] Barile C., Casavola C., Pappalettera G., Kannan V.P. (2022). Damage Progress Classification in AlSi10Mg SLM Specimens by Convolutional Neural Network and K-Fold Cross Validation. Materials.

[33] Iandola F.N., Han S., Moskewicz M.W., Ashraf K., Dally W.J., Keutzer K. (2016). SqueezeNet: AlexNet-Level Accuracy with 50x Fewer Parameters and <0.5 MB Model Size. arXiv.

[34] Mohammadi R., Najafabadi M.A., Saeedifar M., Yousefi J., Minak G. (2017). Correlation of Acoustic Emission with Finite Element Predicted Damages in Open-Hole Tensile Laminated Composites. Compos. B Eng..

[35] Ativitavas N., Pothisiri T., Fowler T.J. (2006). Identification of Fiber-Reinforced Plastic Failure Mechanisms from Acoustic Emission Data Using Neural Networks. J. Compos. Mater..

[36] de Oliveira R., Marques A.T. (2008). Health Monitoring of FRP Using Acoustic Emission and Artificial Neural Networks. Comput. Struct..

[37] Tessema A., Mitchell W., Koohbor B., Ravindran S., van Tooren M., Kidane A. (2018). The Effect of Nano-Fillers on the in-Plane and Interlaminar Shear Properties of Carbon Fiber Reinforced Composite. J. Dyn. Behav. Mater..

[38] Jarrah M., Najafabadi E.P., Khaneghahi M.H., Oskouei A.V. (2018). The Effect of Elevated Temperatures on the Tensile Performance of GFRP and CFRP Sheets. Constr. Build. Mater..

[39] Wisnom M.R., Jones M.I. (1994). A Comparison between Interlaminar and In-Plane Shear Strength of Unidirectional Glass Fibre-Epoxy. Adv. Compos. Lett..

